# Prognostic value of mechanical dyssynchrony in patients with heart failure: a systematic review

**DOI:** 10.1186/s12872-024-04360-6

**Published:** 2024-11-26

**Authors:** Ziqi Chen, Qiang Qu, Iokfai Cheang, Xinyi Lu, Shengen Liao, Rongrong Gao, Yanli Zhou, Xinli Li

**Affiliations:** State Key Laboratory for Innovation and Transformation of Luobing Theory, Department of Cardiology, the First Affiliated Hospital of Nanjing Medical University, Nanjing, 210029 China

**Keywords:** Gated SPECT MPI, Heart failure, Mechanical dyssynchrony, Prognostic analysis

## Abstract

**Background:**

Heart failure (HF) significantly impacts quality of life and healthcare systems worldwide. Assessing left ventricular mechanical dyssynchrony (LVMD) is crucial for understanding cardiac function and optimizing treatments like cardiac resynchronization therapy (CRT). Phase analysis using gated single-photon emission computed tomography (SPECT) myocardial perfusion imaging (MPI) has shown promise in predicting outcomes, yet recent comprehensive reviews are lacking.

**Objective:**

To systematically assess the prognostic value of phase analysis by gated SPECT MPI in the HF population through a systematic review.

**Methods:**

We conducted a systematic review by collecting studies from databases including PubMed, CINAHL, and Web of Science. Two reviewers independently performed study selection, data extraction, and risk of bias assessment. Systematic reviews were conducted using Review Manager Software 5.4 and STATA 16.0.

**Results:**

A total of 2004 patients from seven studies were included in our review and analysis. The systematic review indicated that patients with predetermined clinical events had higher PSD [MD = 6.45, 95% CI (5.83, 7.07), *p* < 0.00001] and PBW [MD = 7.91, 95% CI (5.64, 10.19), *p* < 0.00001]. The diagnosis of LVMD determined by PSD [HR = 1.05, 95% CI (1.01, 1.08), *p* = 0.007] was a strong predictor of endpoint events compared to PBW [HR = 1.95, 95% CI (0.48, 7.89), *p* = 0.35].

**Conclusions:**

The analysis demonstrated that phase information obtained from gated SPECT MPI is of significant prognostic value in patients with heart dysfunction. It effectively enhances clinical risk models, providing reliable guidance for patient treatment.

**Supplementary Information:**

The online version contains supplementary material available at 10.1186/s12872-024-04360-6.

## Introduction

Heart failure (HF) is one of the most severe global health problems, defined as a clinical syndrome with symptoms and/or signs resulting from structural and/or functional cardiac abnormality [1]. As a growing global epidemic, HF not only reduces patients’ quality of life but also presents serious clinical challenges due to its high risk of recurrent hospitalization and mortality. Moreover, it places a substantial economic burden on healthcare systems worldwide [2] According to statistics, HF affects millions of people worldwide, with an incidence estimated to be generally 0.7–8.1 cases per 1000 person-year [3].

Left ventricular mechanical dyssynchrony (LVMD) refers to the mismatch timing of mechanical contraction or relaxation between different segments of the left ventricle (LV) [4]. LVMD is a valuable marker for risk stratification and is associated with worse cardiac structure and function, with prognostic utility in HF patients. Meanwhile, cardiac resynchronization therapy (CRT), directed against dyssynchrony-related events, has been shown to reduce morbidity and mortality in HF [5]. Given this evidence, timely and accurate diagnosis of LVMD in HF patients is of critical clinical importance.

Mechanical dyssynchrony can be evaluated using parameters from conventional M-mode or Doppler echocardiography, but these methods have been insufficient in reliably predicting positive responses to CRT [6]. Similarly, electrical dyssynchrony, diagnosed by prolonged QRS duration, does not accurately reflect the presence of LVMD [7], with only about 70% of patients meeting the QRS duration criteria benefiting from CRT [8]. Recently, indices such as phase standard deviation (PSD) and phase bandwidth (PBW),, derived from phase analysis using gated single-photon emission computed tomography (SPECT) myocardial perfusion imaging (MPI) have been used to evaluate mechanical dyssynchrony comprehensively [9, 10]. Compared to the high variability and unreliability of classical indicators, phase information may serve as an independent predictive factor for cardiac clinical events and be integrated for effective risk stratification [11], aiding in clinical decision-making [12, 13].

However, recent clinical practices and findings regarding the use of SPECT MPI for analyzing survival among HF patients have not been systematically reviewed. This systematic review aims to comprehensively search for and summarized evidence to provide updated references for clinical practice.

## Methods

### Search strategy

This systematic review was conducted in accordance with the Preferred Reporting Items for Systematic Reviews and Meta-Analyses (PRISMA) guidelines [14].

Databases PubMed, CINAHL, and Web of Science were searched from January 1, 2014, to July 1, 2024. Only articles in the English were considered. The MeSH search strategy included the following keywords: “single-photon emission computed tomography,” “SPECT,” “myocardial perfusion imaging,” “MPI,” and “heart failure,” “HF.” The bibliographies of obtained articles were also screened for additional eligible literature. The study included patients who were diagnosed with symptomatic heart failure and had an echocardiographic left ventricular ejection fraction (LVEF) of less than 50%, with gated SPECT MPI (stress/rest protocols) being part of their assessment for left ventricular function.

### Studies were excluded if they met the following criteria:

- ① Presented as oral presentations, poster presentations, systematic reviews, animal studies or case reports.
- ② Repeated publications.
- ③ Studies of incomplete data or full text.
- ④ Inappropriate participant population and designs.
- ⑤ Study with less than 10 patients included and/or 10 assessed diagnostic performance.

### Risk of bias assessment

Two authors (Z.C. and Q.Q.) independently evaluated the methodological quality and risk of bias of the included studies, with any disagreements resolved through discussion with a third party. The Newcastle–Ottawa Scale [15] was used to assess the risk of bias by determining the quality of the observational studies selected, using two independent scales (for cohort and case–control studies). The scale consists of items divided into three domains: selection, comparison, and exposure (for case–control studies) or outcome (for cohort studies). Studies with a rating of 6 or higher were considered high quality.

### Data analysis

RevMan 5.4 software and Stata 16.0 were used for systematic review and assessing bias. Binary variables were statistically analyzed by hazard ratio (HR), and continuous variables were analyzed by mean difference (MD). Each effect size was evaluated with a 95% confidence interval (CI). Statistical heterogeneity was measured using the I^2^ value, and the results are presented in forest plots. The random effects model was used for analysis if I^2^ ≥ 50%; otherwise, the fixed effects model was used. Sensitivity analysis or subgroup analysis was performed to explore potential sources of heterogeneity. Publication bias was assessed using funnel plots and Egger’s test if at least five studies were included in the review.

## Results

### Study selection

**Fig. 1:**
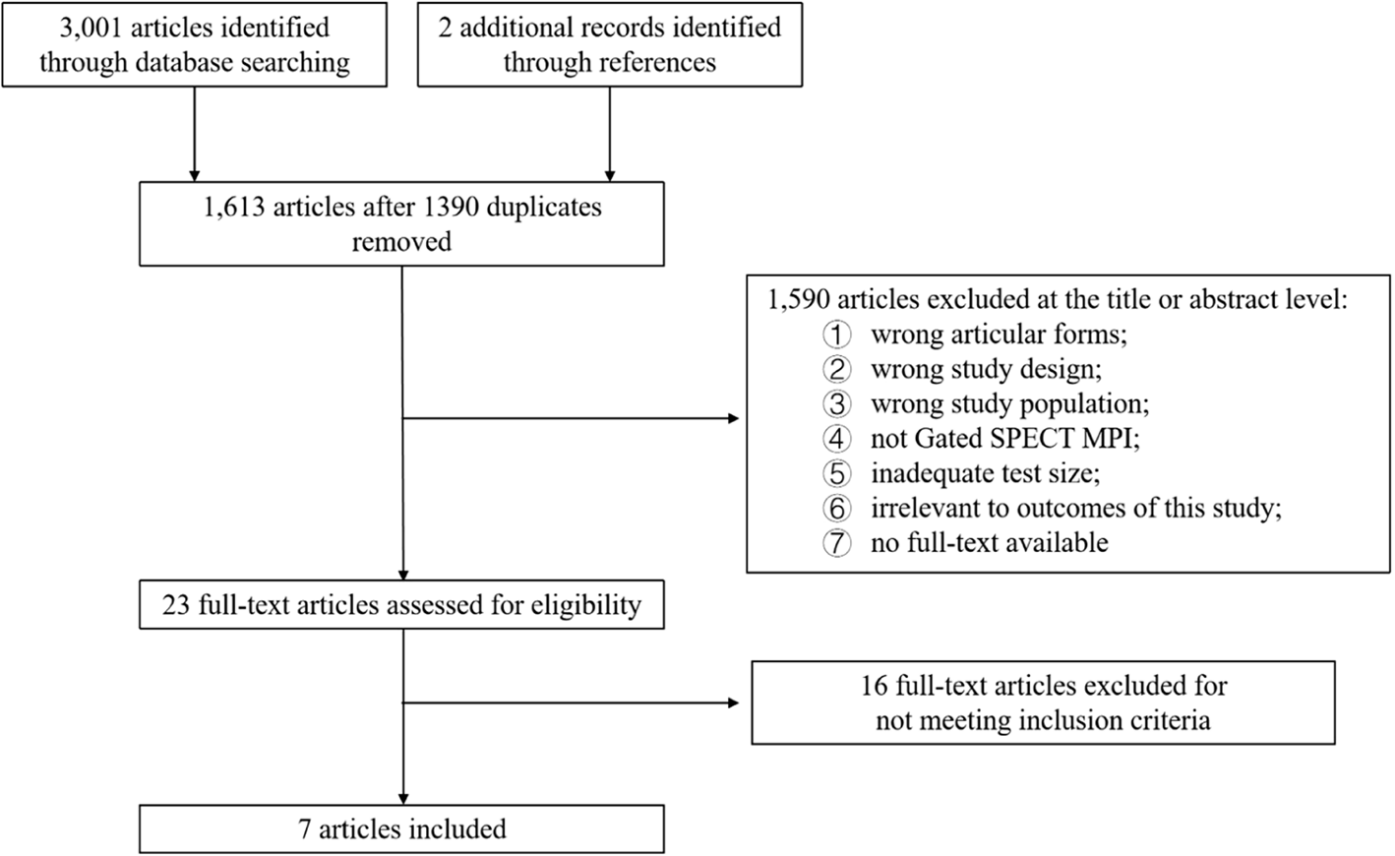
Flow chart of study searching and selection

Figure 1 shows that a total of 3,003 studies were retrieved from the three databases. After removing 1,390 duplicates, 1,613 articles remained for title and abstract screening. Subsequently, 1,590 studies were excluded for reasons such as incorrect article format, not using gated SPECT MPI, inappropriate study design, incorrect study population, inadequate sample size, irrelevant outcomes, or lack of full text. After a full-text review, 16 studies were further excluded for not meeting the inclusion criteria. Ultimately, 7 studies were included in the final review (Supplementary References).

### Study characteristics and grade quality assessment

A total of 2,004 patients, ranging from 35 to 829 per study, were included in our analysis (Table 1). Men constituted the majority of the study population (1,513/2,004).

**Table 1:** Basic characteristics of included studies

Author Year	Study design	Group Size	Age (Mean ± 2SD, yrs)	NYHA	LVEF (%)	Tracer	Indexes	Follow-up (Mean ± 2SD, months)	End Points
Amalia Peix 2014 [16]	P	165	61 ± 9	II, III	< 40%	99mTc-Sestamibi	①②③④⑤⑥	39	HF progression, potentially life-threatening arrhythmic event, cardiac death, non-cardiac death, acute coronary syndrome
Fadi G 2014 [17]	R	170	NR	II, III	≤ 35%	99mTc-Tetrofosmin	①②⑥	24	Sudden cardiac death, fatal MI, and potentially life-threatening arrhythmic event
Nili Zafrir 2017 [18]	P	143	68.3 ± 11	NR	≤ 35%	99mTc-Sestamibi	①②③④⑤⑥⑦⑧	23.7 ± 12.1	Cardiac death, HF exacerbation requiring hospitalization, appropriate VT/VF
Takahiro Doi 2018 [19]	R	570	67.0 ± 12.6	I, II、II, IV	< 50%	99mTc-Tetrofosmin	①③④⑥	19.6	Sudden cardiac death, death due to pump failure and appropriate ICD shock against life-threatening ventricular tachyarrhythmias
Takahiro Doi 2022 [20]	R	829	67.3 ± 12.1	I, II, III, IV	< 50%	99mTc-Tetrofosmin	①③④⑥⑦	37 ± 16	Sudden cardiac death, death due to pump failure, lethal ventricular tachyarrhythmias, and appropriate ICD shock
Yanli Zhou 2021 [21]	P	92	54.6 ± 16	II, II, IV	NR	99mTc-Sestamibi	①②③④⑥⑦⑪	28 ± 10	All-cause death or heart transplantation
Jimmy Bazzy 2021 [22]	R	35	65.1 ± 13.3	III, IV	< 35%	99mTc-Sestamibi	①②⑥⑦	49.2 ± 34.8	All-cause death

①PSD, ②PBW, ③EDV, ④ESV; ⑤SV; ⑥LVEF; ⑦QRS width; ⑧lschemia size; ⑨LVW; ⑩LVMI; ⑪ Rest scar

*PSD*, phase standard deviation, *EDV* End-systolic volume, *ESV* End-systolic volume, *SV* Stroke volume, *LVMI* Left ventricular mass index, *LVEF* Left ventricular ejection fraction, *LBBB* Left bundle branch block, *LVW* Left ventricular weight, *NR* not report, *P* prospective, *R* retrospective

Data were extracted on the first author, publication year, study design, group size, examination indexes, receptor-specific radiotracer, electrocardiographic and echocardiographic markers, SPECT MPI indexes, follow-up time, and corresponding endpoints. Among the selected trials, 4 were performed retrospectively [17, 19, 20, 22]. In five trials [16, 18, 21, 22], patients were injected with the tracer 99mTc-Sestamibi during Gated SPECT MPI, while in the other two trials, 99mTc-Tetrofosmin was used.

Five studies [16, 17, 19–21] focused on the prognostic value of mechanical dyssynchrony diagnosed by SPECT MPI in all patients with HF, and the remaining two emphasized indexes for predicting survival in patients with HF undergoing implantable cardioverter-defibrillator (ICD) or cardiac resynchronization therapy (CRT). The median Newcastle–Ottawa Scale (NOS) scores for the 7 studies was 8 (range: 7–9; Table 2), indicating high quality studies.

**Table 2:** The Newcastle–Ottawa scale of included studies

Reference	Study design	Selection	Comparability	Exposure/Outcome	Total
Amalia Peix 2014 [16]	Prospective	☆☆☆	☆☆	☆☆	☆☆☆☆☆☆☆
Fadi G 2014 [17]	Retrospective	☆☆☆☆	☆☆	☆☆☆	☆☆☆☆☆☆☆☆☆
Nili Zafrir 2017 [18]	Prospective	☆☆☆	☆☆	☆☆☆	☆☆☆☆☆☆☆☆
Takahiro Doi 2018 [19]	Retrospective	☆☆☆☆	☆☆	☆☆☆	☆☆☆☆☆☆☆☆☆
Takahiro Doi 2022 [20]	Retrospective	☆☆☆☆	☆☆	☆☆☆	☆☆☆☆☆☆☆☆☆
Yanli Zhou 2021 [21]	Prospective	☆☆☆☆	☆☆	☆☆☆	☆☆☆☆☆☆☆☆☆
Jimmy Bazzy 2021 [22]	Retrospective	☆☆☆	☆☆	☆☆	☆☆☆☆☆☆☆

### Prognostic performance of SPECT MPI modalities

#### Quantitative analysis

**Fig. 2:**
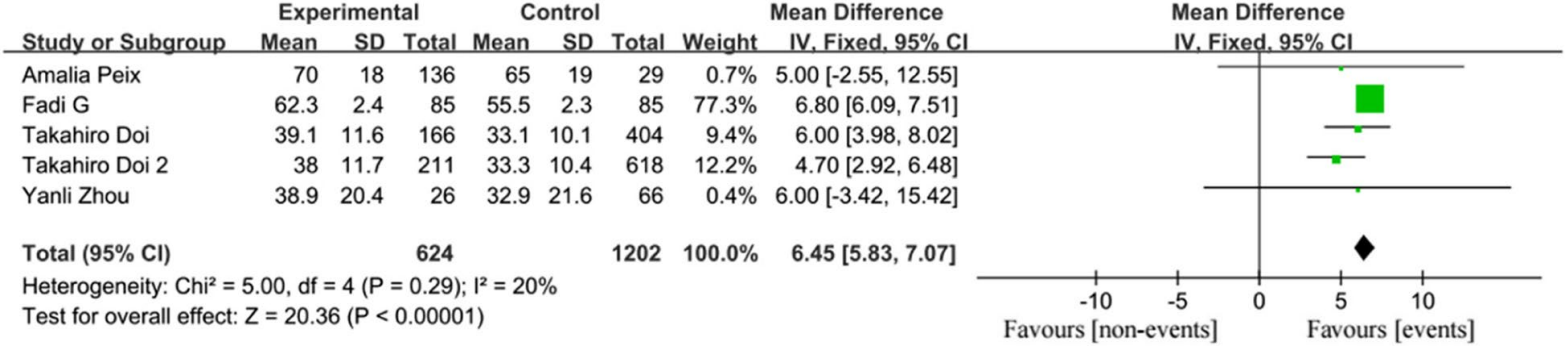
Forest plot of PSD

A total of five studies, including 1,826 patients, reported PSD measurement. The heterogeneity test indicated that the fixed-effects model was appropriate for analysis (I^2^ = 20%, *p* = 0.29). The results showed that the PSD values in the events group was significantly higher than in the non-events group [MD = 6.45, 95% CI (5.83, 7.07), *p* < 0.00001; Fig. 2].

**Fig. 3:**
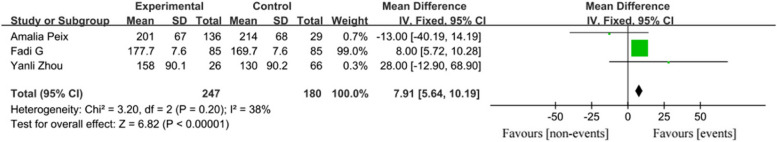
Forest plot of PBW

Three studies, comprising 427 patients, reported PBW degrees. After performing the heterogeneity test, the fixed-effects model was applied (I^2^ = 20%, *p* = 0.38). The results suggested that PBW was significantly higher in the events group compared to the non-events group [MD = 7.91, 95% CI (5.64, 10.19), *p* < 0.00001; Fig. 3].

#### Qualitative analysis

**Fig. 4:**
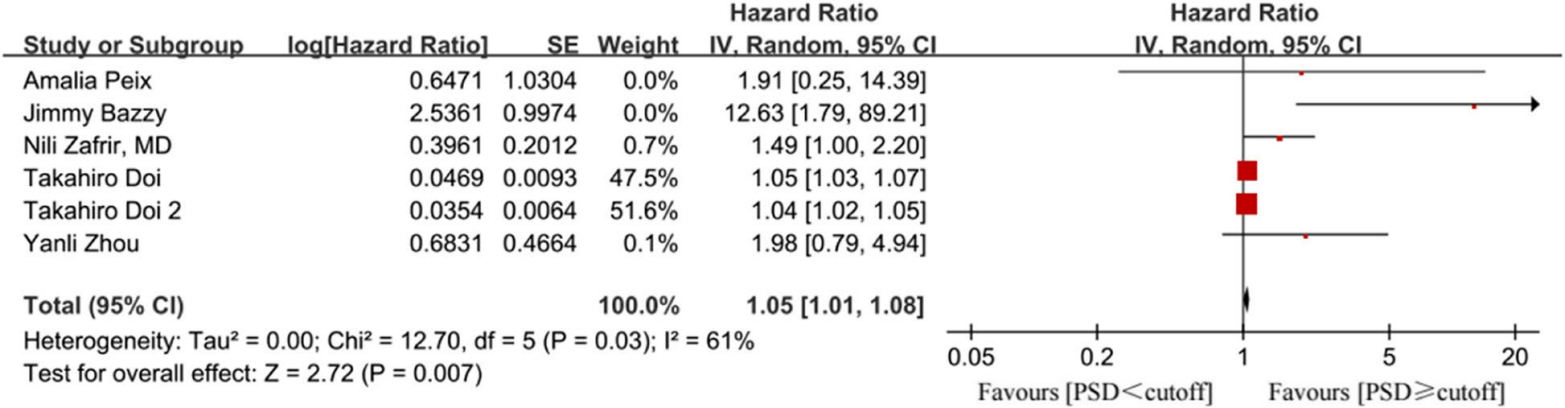
Forest plot of LVMD dependent on PSD

Six studies involving 1,834 patients explored the prognostic value of global LVMD assessed by gated SPECT imaging. Various cutoffs of PSD or PBW were used as thresholds for LVMD. All six articles found that LVMD, as indicated by PSD, was an independent predictor of events. Due to higher heterogeneity (I^2^ = 61%, *p* = 0.03), a random-effects model was used. The analysis showed that the event rate in the experimental cohort was higher than in the control cohort [HR = 1.05, 95% CI (1.01, 1.08), *p* = 0.007; Fig. 4].

**Fig. 5:**
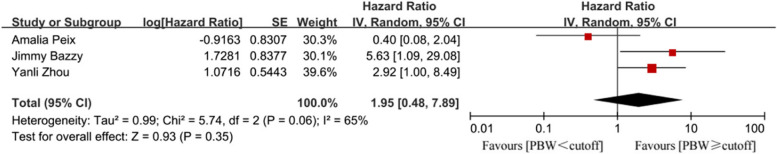
Forest plot of LVMD dependent on PBW

Sensitivity analysis, excluding one study at a time, indicated that the relatively larger sample sizes of two clinical trials [19, 20] contributed to instability. Three studies used PBW cutoffs as an LVMD marker and a random-effects model was used [I^2^ = 65%, *p* = 0.06; Fig. 5]. Results showed no significant difference between groups regarding endpoint events [HR = 1.95, 95% CI (0.48, 7.89), *p* = 0.35]. Sensitivity analysis indicated inconsistency, with HR ranging from 1.19 [95% CI (0.17, 8.29)] to 3.55 [95% CI (1.45, 8.68)]. Details are outlined in the Supplemental materials (Figure S1).

### Publication bias

**Fig. 6:**
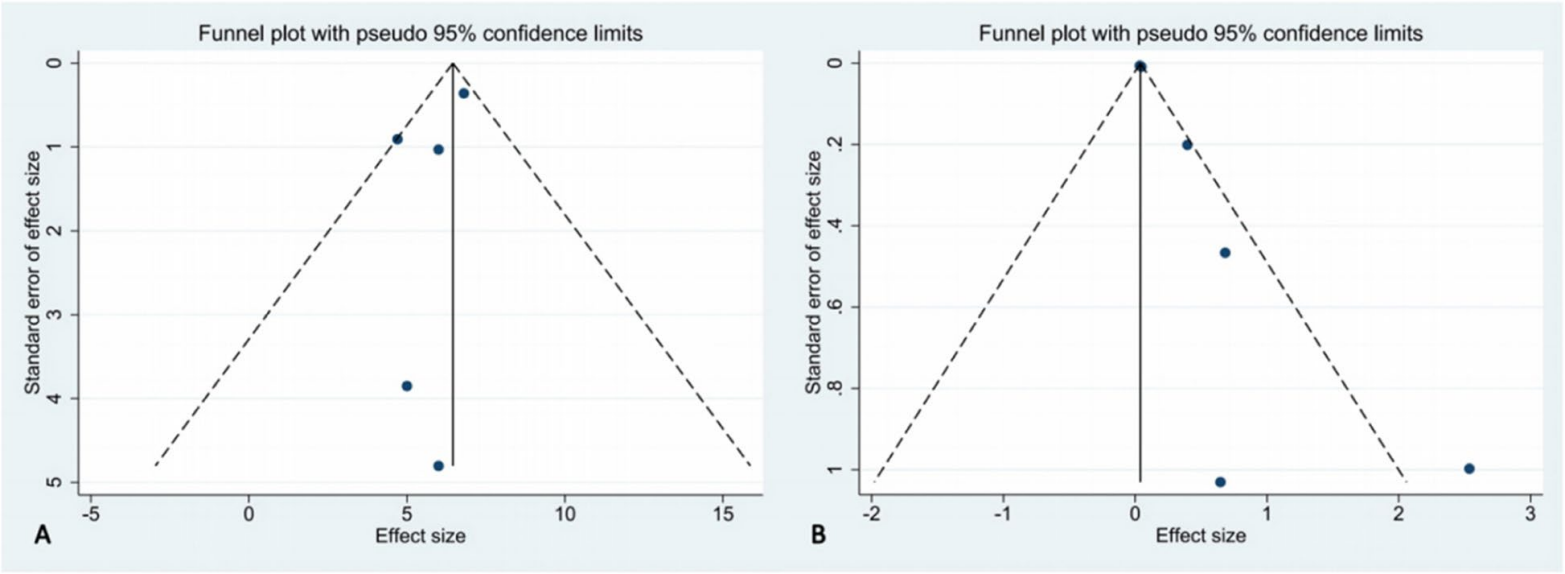
**A** Funnel plots of PSD; **B** Funnel plots of LVMD dependent on PSD

**Fig. 7:**
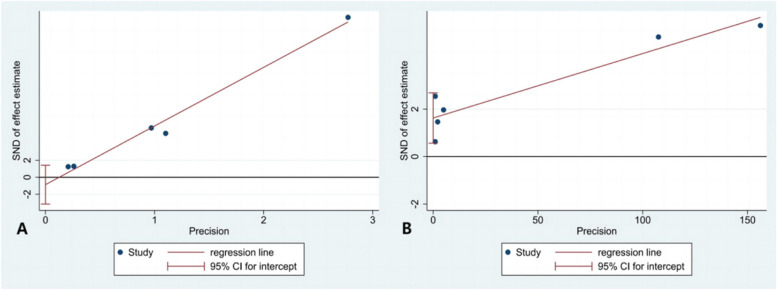
**A** Egger’s tests of PSD; **B** Egger’s tests of LVMD dependent on PSD

STATA16.0 software was used to generate funnel plots (Fig. 6) and conduct Egger’s tests (Fig. 7) to evaluate publication bias. Due to the limited number of studies, We focused on publication biases in the quantitative and qualitative analysis of PSD. The funnel plots showed uneven and asymmetric distributions, but Egger’s test indicated no significant publication bias in the quantitative analysis of PSD (*p* = 0.315, Fig. 7A), while the qualitative analysis showed a potential bias (*p* = 0.013, Fig. 7B).

## Discussion

This systematic review included 7 studies involving 2,004 patients, primarily male, to evaluate the prognostic value of mechanical dyssynchrony using gated SPECT MPI in HF patients. Quantitative analyses showed that patients with adverse events had significantly higher PSD and PBW values. The results suggested that PSD is a consistent predictor of adverse cardiac outcomes, while the predictive value of PBW was less conclusive due to higher variability.

Cardiac dyssynchrony, encompassing both electrical and mechanical components, reflects diminished ventricular function, contributing to worsened outcomes in HF patients [23], with electrical dyssynchrony commonly manifesting as QRS prolongation [24], and closely associated with reduced LVEF and elevated cardiac mortality in patients with HF [25]. Although electrical dyssynchrony is a key criterion for CRT, many patients do not respond adequately, highlighting the need for additional parameters beyond QRS duration and LVEF for optimal patient selection [6].

LVMD involves asynchronous contraction of LV segments, directly impacting cardiac remodeling and ejection function. Notably, mechanical dyssynchrony can be present even in patients with normal or mildly reduced LVEF and narrow QRS complexe [26, 27]. Historically, LVMD was diagnosed using M-mode echocardiography by comparing the interval between posterior wall contraction and the rapid filling phase [28]. Tissue Doppler echocardiography has since evolved, enabling the measurement of peak systolic and diastolic velocities, as well as the amplitude and initiation times of these phases. Newer modalities include tissue Doppler imaging (TDI), strain rate imaging (SRI), tissue synchronization imaging (TSI), and magnetic resonance imaging (MRI) [4].

**Fig. 8:**
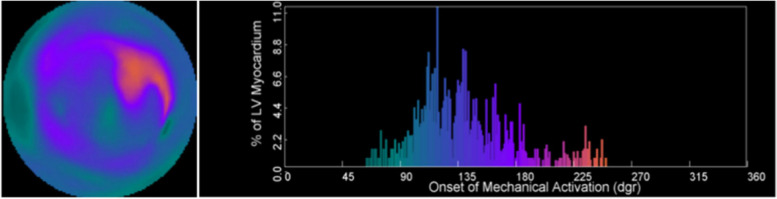
An example of a 64 years old male patient with AHF (LVEF = 46.8%) died after followed up 22 months. PBW (164°) was measured by the phase analysis of SPECT MPI. Reprinted with permission from ref. 24. Copyright (2021) Journal of Nuclear Cardiology

Among nuclear imaging techniques, SPECT myocardial perfusion imaging (MPI) is widely used to generate continuous 3D images of the ventricle, aligning with specific phases of the cardiac cycle to visualize myocardial perfusion. In clinical practice, SPECT is frequently applied for the diagnosis and risk stratification of coronary artery disease. Complementary software has been developed to extract phase information from SPECT, such as standard deviation (SD) and phase bandwidth (PBW), which are increasingly used to assess myocardial mechanical motion and diagnose LVMD (Fig. 8) [21].

Pazhenkottil et al. [29] concluded that LVMD, evaluated through phase analysis, serves as a strong independent predictor of clinical events, regardless of other factors such as perfusion defects or decreased LVEF. This is consistent across various patient groups, including those with coronary artery disease and reduced LVEF without CRT [30], as well as patients with non-ischemic cardiomyopathy, LVEF of 35–50%, and QRS < 150 ms [9]. Additionally, several studies have suggested that LVMD, defined by PSD, is an effective biomarker for predicting outcomes post-CRT [6, 9, 19, 26]. Consistent with these findings, our systematic review found that patients with endpoint events had significantly higher PSD and PBW values, and the PSD cut-off point played a significant role in distinguishing outcomes.

The phase information derived from SPECT MPI reflects three-dimensional heterogeneity in myocardial perfusion abnormalities, cardiomyocyte injury, and metabolic impairment, contributing to electrophysiological instability, arrhythmias, and decreased contractility [13, 19]. Combining LVMD with other factors such as the heart-to-mediastinum ratio (HMR), LVEF, QRS duration, and left ventricular mass index (LVMI) further enhances its prognostic value in HF risk models [20, 31]. Moreover, the fully automated nature of phase analysis using SPECT MPI enhances reproducibility and reduces operator-dependent variability, offering a more standardized approach to diagnosing LVMD. This can improve the accuracy of risk assessments, guiding personalized treatment strategies. Additionally, the cost-effectiveness and reduced time burden of SPECT MPI compared to other imaging modalities, such as MRI, make it a practical choice for routine clinical use, especially in resource-constrained settings.

While echocardiography is highly dependent on the operator’s expertise, leading to variability in results and limited reproducibility, MRI presents disadvantages such as low temporal resolution, lengthy procedures, and high costs [4]. Conversely, the automated determination of PSD from SPECT MPI minimizes manual errors during data acquisition, saving both time and cost. Furthermore, retrospective indexing from previous records also avoids the need for repeated imaging and testing. This systematic review supports the use of gated SPECT MPI parameters, particularly PSD and PBW, as robust tools for risk stratification and prognostication in HF patients. The findings underscore the importance of integrating mechanical dyssynchrony measures with traditional risk factors to enhance HF management and patient outcomes.

## Strengths and limitations

The strengths of this systematic review include its adherence to PRISMA guidelines, a comprehensive search strategy, and rigorous inclusion and exclusion criteria, all of which contribute to the reliability of the findings. However, significant heterogeneity among studies, particularly in hazard ratios, may be attributed to variations in patient populations and different methods for determining cutoff values. Notably, only one study [17] used propensity score matching to ensure well-balanced baseline characteristics among subjects. Other studies categorized patients based on endpoint events without accounting for variables such as etiology, clinical characteristics, or the use of mortality-reducing drugs.

Also, the prevalence of ischemic coronary disease, diabetes mellitus, and other comorbidities related to dyssynchrony was a crucial factor influencing mechanical contraction patterns [32] and the development of clinical events [16]. More subgroup analyses are necessary to validate these conclusions. The determination of cutoff values for PSD and PBW varied across studies, especially in defining dyssynchrony for comparison. Some studies employed receiver operating characteristic (ROC) curve analysis to identify optimal cutoffs [16, 19, 20], while others used the mean ± 2 × SD from a control group as the LVMD threshold [18, 21]. One study [22] did not provide information on the origin of its cutoff value.

Furthermore, differences in the setting of endpoint events, along with variations in experimental design, likely contribute to instability in survival analysis. For instance, Kaplan–Meier survival curves related to the PSD cutoff in Nili Zafrir [27] showed differentiation in cardiac death and HF deterioration but a similar cumulative event rate of ventricular tachycardia or ventricular fibrillation following CRT or ICD. Moreover, substantial diversity could be observed within nuclear tracer, injection dosage, time interval before imaging, gamma camera, radiotracer and gated SPECT software. Most of the articles enrolled in our systematic review were single-center observational studies with relatively small population. More large-scale, rigorously conducted prospective multicenter studies are needed to provide high-quality evidence for determining cutoff value for diagnosing LVMD and establishing a risk-based therapeutic strategy using SPECT MPI [33].

## Conclusion

SPECT MPI phase analysis offers incremental prognostic value in HF patients, particularly when combined with traditional markers such as LVEF and QRS duration. Future high-quality multicenter studies are essential to confirm these findings and establish evidence-based guidelines for integrating SPECT MPI in clinical practice.

## Supplementary Information

Supplementary Material 1.

## Data Availability

No datasets were generated or analysed during the current study.
