# Supplementary material for: Prognostic value of mechanical dyssynchrony in patients with heart failure: a systematic review
Source: BMC Cardiovasc Disord. 2024 Nov 26;24:674. doi: 10.1186/s12872-024-04360-6 (PMC11590517; doi:10.1186/s12872-024-04360-6)
Supplement: Supplementary file 1 — Supplementary Material 1. [file 12872_2024_4360_MOESM1_ESM.docx]

**Supplemental Material**

1. **Supplementary Figure S1**

①Sensitivity analysis of LVMD dependent on PSD

**
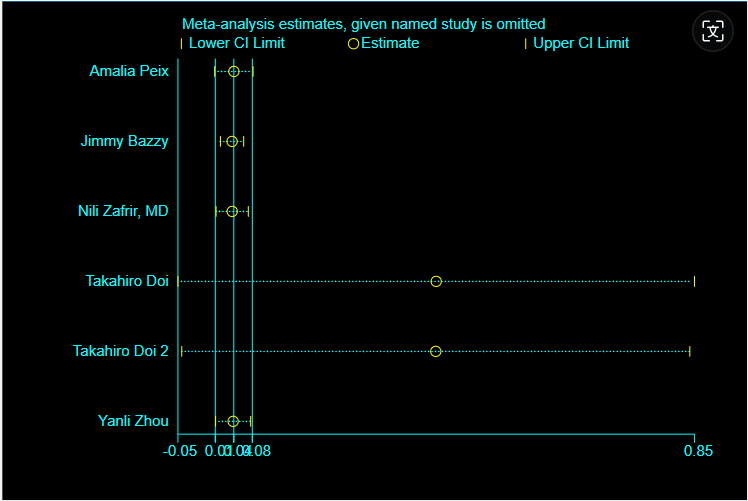
**

②Sensitivity analysis of LVMD dependent on PBW


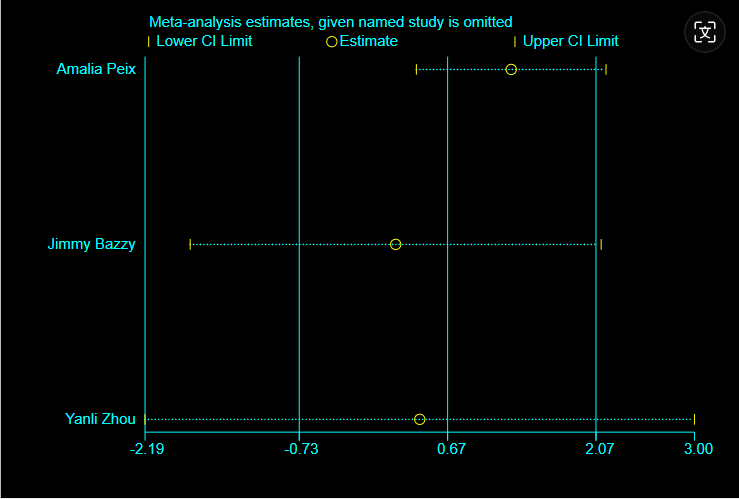


**2. Supplementary References**

1. Bazzy J, Gadiyaram V, Nguyen BJ, Birgersdotter-Green U, Hoh CK, Obrzut S: **Gated single-photon emission computed tomography myocardial perfusion imaging phase analysis as an imaging biomarker for mortality prediction in heart failure patients undergoing cardiac resynchronization therapy**. *Nucl Med Commun* 2021, **42**(9):990-997.

2. Doi T, Nakata T, Noto T, Mita T, Yuda S, Hashimoto A: **Improved risk-stratification in heart failure patients with mid-range to severe abnormalities of QRS duration and systolic function using mechanical dyssynchrony assessed by myocardial perfusion-gated SPECT**. *J Nucl Cardiol* 2022, **29**(4):1611-1625.

3. Doi T, Nakata T, Yuda S, Hashimoto A: **Synergistic prognostic implications of left ventricular mechanical dyssynchrony and impaired cardiac sympathetic nerve activity in heart failure patients with reduced left ventricular ejection fraction**. *Eur Heart J Cardiovasc Imaging* 2018, **19**(1):74-83.

4. Hage FG, Aggarwal H, Patel K, Chen J, Jacobson AF, Heo J, Ahmed A, Iskandrian AE: **The relationship of left ventricular mechanical dyssynchrony and cardiac sympathetic denervation to potential sudden cardiac death events in systolic heart failure**. *J Nucl Cardiol* 2014, **21**(1):78-85.

5. Peix A, Karell J, Rodriguez L, Cabrera LO, Padron K, Carrillo R, Mena E, Fernandez Y: **Gated SPECT myocardial perfusion imaging, intraventricular synchronism, and cardiac events in heart failure**. *Clin Nucl Med* 2014, **39**(6):498-504.

6. Zafrir N, Bental T, Strasberg B, Solodky A, Mats I, Gutstein A, Kornowski R: **Yield of left ventricular dyssynchrony by gated SPECT MPI in patients with heart failure prior to implantable cardioverter-defibrillator or cardiac resynchronization therapy with a defibrillator: Characteristics and prediction of cardiac outcome**. *J Nucl Cardiol* 2017, **24**(1):122-129.

7. Zhou Y, He Z, Liao S, Liu Y, Zhang L, Zhu X, Cheang I, Zhang H, Yao W, Li X *et al*: **Prognostic value of integrative analysis of electrical and mechanical dyssynchrony in patients with acute heart failure**. *J Nucl Cardiol* 2021, **28**(1):140-149.
